# Histone Lysine Demethylase JMJD2D/KDM4D and Family Members Mediate Effects of Chronic Social Defeat Stress on Mouse Hippocampal Neurogenesis and Mood Disorders

**DOI:** 10.3390/brainsci10110833

**Published:** 2020-11-09

**Authors:** Swati Maitra, Nitin Khandelwal, Scherazad Kootar, Pooja Sant, Salil S. Pathak, Sujatha Reddy, Annapoorna P. K., Upadhyayula Suryanarayana Murty, Sumana Chakravarty, Arvind Kumar

**Affiliations:** 1 Applied Biology, CSIR—Indian Institute of Chemical Technology (IICT), Uppal Road, Tarnaka, Hyderabad 500007, Telangana, India; maitra.swat@gmail.com (S.M.); murtyusn@gmail.com (U.S.M.); 2 Epigenetics & Neuropsychiatric Disorders Laboratory, CSIR—Centre for Cellular and Molecular Biology (CCMB), Uppal Road, Habsiguda, Hyderabad 500007, Telangana, India; srnphkhandelwal@gmail.com (N.K.); k.scherazad@gmail.com (S.K.); poojasant83@gmail.com (P.S.); salilsaurav@gmail.com (S.S.P.); sujathaccmb@gmail.com (S.R.); annapoornap@ccmb.res.in (A.P.K.); 3 Academy of Scientific and Innovative Research (AcSIR), Ghaziabad 201002, Uttar Pradesh, India; 4 National Institute of Pharmaceutical Education and Research (NIPER), Guwahati 781101, Assam, India

**Keywords:** jumonji domain-containing histone demethylases, Dimethyloxallyl glycine (DMOG), epigenetic regulators, chromatin modifications, dentate gyrus (DG), neural stem or progenitor cells (NSCs/NPCs), H3K9me2/3, neurosphere culture

## Abstract

Depression, anxiety and related mood disorders are major psychiatric illnesses worldwide, and chronic stress appears to be one of the primary underlying causes. Therapeutics to treat these debilitating disorders without a relapse are limited due to the incomplete molecular understanding of their etiopathology. In addition to the well-studied genetic component, research in the past two decades has implicated diverse epigenetic mechanisms in mediating the negative effects of chronic stressful events on neural circuits. This includes the cognitive circuitry, where the dynamic hippocampal dentate gyrus (DG) neurogenesis gets affected in depression and related affective disorders. Most of these epigenetic studies have focused on the impact of acetylation/deacetylation and methylation of several histone lysine residues on neural gene expression. However, there is a dearth of investigation into the role of demethylation of these lysine residues in chronic stress-induced changes in neurogenesis that results in altered behaviour. Here, using the chronic social defeat stress (CSDS) paradigm to induce depression and anxiety in C57BL/6 mice and *ex vivo* DG neural stem/progenitor cell (NSCs/NPCs) culture we show the role of the members of the JMJD2/KDM4 family of histone lysine demethylases (KDMs) in mediating stress-induced changes in DG neurogenesis and mood disorders. The study suggests a critical role of JMJD2D in DG neurogenesis. Altered enrichment of JMJD2D on the promoters of *Id2* (inhibitor of differentiation 2) and *Sox2* (SRY-Box Transcription Factor 2) was observed during proliferation and differentiation of NSCs/NPCs obtained from the DG. This would affect the demethylation of repressive epigenetic mark H3K9, thus activating or repressing these and possibly other genes involved in regulating proliferation and differentiation of DG NSCs/NPCs. Treatment of the NSCs/NPCs culture with Dimethyloxallyl Glycine (DMOG), an inhibitor of JMJDs, led to attenuation in their proliferation capacity. Additionally, systemic administration of DMOG in mice for 10 days induced depression-like and anxiety-like phenotype without any stress exposure.

## 1. Introduction

Major depressive disorder is a chronic, debilitating mental illness that is rising globally and its predominant cause appears to be chronic or persistent stress [1,2,3]. The available medications to treat this condition act quite slowly, taking at least a month to improve an individual’s mood. Moreover, these medications fail to prevent relapse and cause innumerable side effects in responding individuals [4,5,6]. Therefore, there is an urgent need for new therapeutics, which is possible only when the molecular mechanisms in the etiopathology of the disease are well understood.

Chronic stress has negative effects on multiple brain regions and one of the regions severely affected is the dentate gyrus (DG), the neurogenic niche in the hippocampus, where adult neurogenesis occurs [7,8,9,10]. Evidence for adult neurogenesis even in old age has been found in rodents, nonhuman primates, and recently, in humans too [11,12]. Factors such as stress, anxiety, depression, addiction, etc. work as impairers, whereas; exercise, antidepressants, antipsychotics, electroconvulsive therapy etc. act as enhancers of the neurogenesis [13,14,15,16,17]. A large number of preclinical and clinical research reports have focused on the interactions between stress and depression and their effects on various brain regions such as nucleus accumbens (NAc), prefrontal cortex (PFC), hippocampus, etc. [18,19,20,21]. Depression and related affective disorders alter both the mesolimbic reward circuitry, which involves the NAc; and the cognitive circuitry, which involves the hippocampus and its neurogenic niche, the DG [22,23,24]. The cognitive circuitry of the brain gets affected by chronic perturbations of the nervous system, which are triggered by diverse psychosocial stressors. Stress leads to the dysregulation of several genes through modifications in the epigenetic or chromatin remodelling events in and around their regulatory regions [21,25,26]. Therapeutics such as antidepressants and electroconvulsive therapy (ECT) also appear to act by altering neural epigenetic mechanisms [27,28] in the restoration of the affected hippocampal neurogenesis or its enhancement [29,30].

Moreover, using rodent models of depression and/or anxiety, numerous studies have shown that the deleterious effects of repeated stress on both reward and cognitive circuitry are mediated by diverse epigenetic mechanisms [21,31]. Many of these studies have focused on histone lysine acetylation/deacetylation, which is correlated with transcriptional activation/repression, respectively [21]. In addition, few studies have also implicated histone lysine methylation in the affected brain circuitries of animal models of depression. For instance, Nestler’s group reported an increase in transcriptionally repressive H3K9/K27 dimethylation at hundreds of gene promoters in the NAc of defeated mice. Interestingly, the hypermethylation on many of these promoters was reversed by the systemic administration of imipramine, a commonly used antidepressant, for a month [32]. Alterations in histone-modifying enzymes were also reported in various brain regions of rats and mice when they were exposed to social defeat stress [33,34]. Transcriptional repression of the critical brain-derived neurotrophic factor (*Bdnf*) has been reported in C57BL/6 mice exhibiting depression-like phenotype following chronic social defeat stress (CSDS). This correlated well with increased histone H3K27 dimethylation on its promoter III and IV. Administration of imipramine for a month improved the mood of the mice, along with an increase in transcriptionally activating epigenetic marks, H3K9 and H3K14 acetylation, and H3K4 trimethylation, on *Bdnf* promoters [27]. Overexpression of one of the histone deacetylases (HDACs) prevented the therapeutic effects of imipramine by reducing H3 acetylation. Nonetheless, this study looked into the whole hippocampus and not exclusively in the DG, where neurogenesis is reported to be affected in chronic stress-induced animal models of depression [35,36,37].

Studies have also indicated the involvement of epigenetic mechanisms such as histone acetylation and deacetylation in mediating the effects of chronic stressful events on adult neurogenesis in mouse models [38,39,40]. Few studies have implicated H3K9 methylation in stress-induced changes in DG neurogenesis as well [31,41]. The role of histone methylases and demethylases in Embryonic Stem Cell (ESC) proliferation and their regulation upon differentiation has also been addressed [42,43]. However, the role of histone lysine demethylases, especially those that regulate H3K9 and H3K27 demethylation, is hardly explored in the context of stress-induced neuroglial changes in the cognitive circuitry and in adult DG neurogenesis. These modifications are involved in transcriptional repression and few studies have shown an association of some of the histone demethylases with the neural stem cell (NSC) proliferation and differentiation [44,45]. In one report, jumonji domain-containing histone lysine demethylases (JMJDs) such as JMJD3 and its target H3K27me2/3 have been shown to regulate NSC biology [46].

In one of our previous collaborative works [47], three weeks of postnatal maternal separation stress has been shown to induce diverse mood and cognitive disorder-like phenotype in rats at later stages of life. Here, alteration in *Bdnf* gene function has been reported to be a major underlying cause where H3K9me2/3 and H3K27me2/3 appeared to play a critical role resulting in compromised hippocampal function and DG neurogenesis. Considering these previous studies, which delineate the importance of repressive H3K9 and H3K27 methylation marks in mediating the effects of stress on various brain regions and the dearth of studies on histone demethylases, we focused on a family of lysine demethylases which act on H3K9me2/3.

The present study aims to uncover the role of the KDM4 family, also known as the Jumonji domain-containing histone lysine demethylase 2 (JMJD2) family, in DG neurogenesis in a mouse depression model of CSDS. We report that 10 days of CSDS exposure to C57BL/6 and Nestin-GFP mice (in C57BL/6 and 129 mixed backgrounds) induces mood disorders, affects proliferation of neural stem or progenitor cells (NSCs/NPCs) in DG, and attenuates neurogenesis. Upon mapping of the lysine-specific histone demethylases, we found a significant level of dysregulation in the mRNA levels of KDM4/JMJD2 family members in the DG of the defeated mice. We also observed their differential expression when proliferating neurospheres, established from the NSCs/NPCs were subjected to differentiation. We identify a critical role of JMJD2D in chronic stress-induced changes in DG neurogenesis and propose that uncovering its role in the adult mouse brain in detail might help in devising better therapeutic approaches to treat depression, anxiety, and related mood disorders.

## 2. Materials and Methods

### 2.1. Animals

Male, 6–8 weeks old C57BL/6Ncrl mice and Nestin-GFP transgenic mice (in the C57BL/6 background) were used in all the experiments except the ex-vivo neurosphere cultures, where post- natal day2 old C57BL/6Ncrl male mice were used. Male, 6–8 months old, aggressive, CD1 retired breeders were used as resident mice in the social defeat paradigm. All experiments were performed in accordance with the standard protocols and procedures approved by the Institutional Animal Ethics Committee (IAEC). Throughout the experiment, mice were maintained in a 12 h/12 h light and dark cycle at constant temperature (25 °C) and 60% relative humidity. The mice received standard chow and water ad libitum until specified such as in the sucrose preference test.

### 2.2. Chronic Social Defeat Stress

The 10-day chronic social defeat stress (CSDS) is a well-established paradigm to induce depression-like phenotype in mice, which is analogous to the depression phenotype seen in humans [27]. As described in earlier reports, the experimental mouse was kept with an aggressor CD1 mouse (resident) in the same cage, where both were separated by a perforated, Plexiglas barrier. Daily, the experimental mouse was exposed to the antagonistic behaviour of the resident mouse for 5 min and transferred back to the other half of the cage, where it remains only in sensory contact with the aggressor for the rest of the day [48,49,50]. Throughout the defeat paradigm, the experimental mice were daily exposed to a different, unfamiliar CD1 aggressor to avoid any habituation. Control mice were housed in pairs on either side of the similar cages and were allowed to interact with each other for 5 min, every 24 h. Overall, 30% of the experimental mice did not show defeated or depression-like phenotype after the CSDS protocol and were considered as “resilient”. For the present study, we have specially focused on the correlation between depression-like phenotype and neurogenesis. Therefore, the “resilient” mice were excluded from the study and only the truly defeated mice (based on behavioural parameters) were used for the experiments.

### 2.3. Social Interaction Test

The development of depression-like phenotype was assessed using the well-established social interaction test, which is a measure of interaction and avoidance of the experimental mouse towards the aggressor mouse used in the CSDS paradigm, according to previously reported protocols [48,49,50]. In brief, the experimental mouse was introduced into a novel arena from one end, wherein one unfamiliar aggressor CD1 mouse was kept in a small perforated Plexiglas chamber on the other end. Time spent by the experimental mouse in the interaction zone was measured for 5 min, in the absence (1st session) as well as in the presence (2nd session) of the target CD1 mouse, using Ethovision 3.1 (Noldus, The Netherlands) and interaction ratio was calculated.

### 2.4. Sucrose Preference Test

Sucrose preference test (SPT) is a classic paradigm for assessing anhedonia, a major hallmark of depression in humans. This test was performed according to the published protocols [49,50]. Briefly, mice were given a two-bottle choice between plain water and 2% sucrose solution to drink and their consumption was measured daily throughout the defeat paradigm. Before starting the stress paradigm, all mice were acclimatized for the two-bottle choice condition for at least 4 days. Sucrose preference was determined by calculating the average sucrose solution consumption by mice over the last 4 days of testing. Positions of both the bottles were interchanged every day to eliminate any positional bias.

### 2.5. Tissue Collection for Molecular Studies and qPCR Analysis

Mice were sacrificed and the brain was rapidly removed, placed on a chilled brain matrix to slice into sections of 1 mm thickness. The DG region was microdissected using a 16-gauge needle and snap-frozen in liquid N_2_, later stored in −80 °C until use. RNA was isolated from these tissues using the standard TRIzol method (Invitrogen) and cDNA was synthesized by the first-strand cDNA synthesis method using oligo d(T)_20_ primer and SuperScript™ III Reverse Transcriptase (Invitrogen) enzyme. cDNA samples were amplified for the gene of interest using specific primer sets by qPCR using SYBR Green Master Mix (Takara). All the reactions were set up in triplicates in 384 well optical plates in the *Viia7* real-time PCR machine (Life Technologies) and the data was analysed by the comparative ΔΔCt method and normalized with the *Gapdh* mRNA levels.

### 2.6. Western Blotting

Protein samples were prepared by lysis of both proliferating and differentiating population of NSCs/NPCs in Laemmli buffer, followed by centrifugation and the soluble fractions were used for SDS-PAGE. Resolved proteins were transferred onto PVDF membrane and incubated overnight with respective antibodies in 2.5% BSA at 4 °C after blocking with 5% BSA. After washing with TBS-Tween-20, blots were incubated with appropriate secondary antibodies for 1 h at room temperature and then processed for enhanced chemiluminescence (ECL) detection using Western lighting chemiluminescence reagent from Abcam. The intensity of bands was quantified using ImageJ software. β actin was used as a loading control.

### 2.7. Immunohistochemical Staining on Tissue Sections

Immunohistochemical staining was performed as reported earlier [51] with few modifications as per experimental requirements. For assessment of proliferation, mice received i. p. injection of BrdU (200 mg/kg body weight) before the last stress episode and sacrificed 30 min after the stress for the 0 h group, whereas for the other group, BrdU was injected after the last stress episode and mice were sacrificed 24 h later. In both the groups, mice were intra-cardially perfused with 4% paraformaldehyde (PFA) and the brain was dissected out and preserved in 30% sucrose. 30 µm thick coronal sections were taken from the part containing hippocampus and serially collected in 6 wells; each series contains every 6th section. From each well (series), 8 sections were randomly selected and mounted on positively charged glass slides. Staining for BrdU was performed using SuperPicture IHC detection kit (Invitrogen) as reported previously [52]. In brief, sections were pre-treated with Formamide, hydrolysed using 2 N HCl, followed by neutralization, peroxide quenching, and finally blocking with 10% normal horse serum. Subsequently, sections were incubated overnight with anti-BrdU antibody (1:500, Calbiochem) followed by incubation with antimouse biotin-conjugated secondary antibody (1:200, Vector Labs) for 2 h. Finally, Vectastain ABC system was used to develop signals using 3, 3′ Diaminobenzidine (DAB) as a substrate and DPX as mountant.

For Nestin and DCX staining, blocking was done with 10% normal horse serum and sections were incubated overnight with anti-GFP (for Nestin, 1:100, Santa Cruz, TX, USA) and anti-DCX (1:500, Abcam, Cambridge, UK) antibodies separately. Subsequently, sections were incubated with antimouse FITC (1:1000, Santa Cruz, against GFP for Nestin) and anti-rabbit Alexa Fluor 555 (1:500, Molecular Probes, for DCX) for 2 h, followed by mounting in Vectastain with DAPI.

Cells positive for DCX, Nestin, and BrdU were quantified by stereological cell counting method as explained by a few previous studies [53,54]. In brief, sections were visualized under a fluorescent microscope at 20× magnification, and cells positive for the respective markers were counted manually. Cells from either side of 8 sections from mouse DG were counted and their number was multiplied with the periodicity of sections i.e., 6 to calculate the total no. of positive cells per DG. Representative images were taken at 10× and 20× using a confocal microscope.

### 2.8. Ex Vivo Neurosphere Culture

Hippocampal-derived NSCs/NPCs were cultured according to previous reports [54] with slight modifications [25,51]. Briefly, pups were sacrificed and hippocampi were dissected, minced, and triturated in 0.025% trypsin–EDTA followed by incubation for 7 min at 37 °C. The activity of trypsin was arrested by 0.014% Trypsin inhibitor, containing 1 mg/mL DNase I enzyme. The cell suspension was passed through a 40 μm sieve to obtain single cells and then pelleted at 700× *g* for 5 min. Pelleted cells were resuspended in 1 mL of NeuroCult NSC basal media with 10× mouse proliferative supplement (Stemcell Technologies, 05702), 100 U/mL penicillin, 40 μg/mL streptomycin, 0.02% BSA, 10 ng/mL bFGF, 20 ng/mL EGF, and 0.04 mg/mL heparin by gentle trituration. Cells were counted and accordingly seeded in 96-well plate (0.4 × 10^4^ cells/well @ 2 × 10^4^ cells/mL) and T-25 flasks (1 × 10^5^ cells/flask @ 2 × 10^4^ cells/mL) and allowed to grow into neurospheres. Neurospheres were sub-cultured by dissociation into single-cell suspension using StemPro Accutase Cell Dissociation Reagent (Gibco) and re-plating in the complete NeuroCult proliferation medium.

For differentiation, after 5–6 days of proliferation, neurospheres were dissociated into single-cell suspension using Accutase and plated on 10 cm culture dishes coated with poly-D-lysine (0.1 mg/mL, Sigma) and laminin (10 μg/mL, Gibco) for 2 h each at 37 °C. The differentiation medium comprised of NeuroCult NSC basal medium with 1% fetal bovine serum, 100 U/mL penicillin, 40 µg/mL streptomycin, 0.02% BSA.

Treatment of neurospheres with DMOG (Dimethyloxallyl glycine): NPCs/NSCs were plated @ 2 × 10^4^ cells/mL in 96 well plates (for neurosphere counting) and in 24 well plates (for qPCR studies). DMOG (cat #D1070, Frontier Scientific Inc., Logan, UT, USA) was dissolved in 1× PBS and added daily for 6 consecutive days at different doses (100 µM, 200 µM, and 600 µM) in the respective wells containing the cells. Images of neurospheres were taken on the 7th day under Axiovert Live Cell Imaging Microscope (Zeiss, Oberkochen, Germany) while their diameter was measured under 10× magnification using the AxioVision LE Rel.4.3 by imaging random different fields and were grouped into 3 groups of <50 µm, 50–100 µm, and >100 µm diameter.

### 2.9. Administration of DMOG In Vivo

DMOG, as described in the earlier report [49], dissolved in saline, was administered intraperitoneally (i. p.) in the experimental mice at a dose of 40 mg/kg body weight daily for 10 consecutive days whereas control mice were administered with an equal volume of saline. 

### 2.10. Chromatin Immunoprecipitation and qPCR (ChIP-qPCR)

ChIP assay was performed as published in earlier studies [55,56] with minor modifications. Briefly, both proliferating and differentiating NPCs/NSCs were cross-linked using 1% formaldehyde and sonicated for 90 cycles (30 s OFF/30 s ON) at high amplitude. 30 µg of chromatin samples were incubated with 3 µg of anti-rabbit JMJD2d antibody at 4 °C for 12–13 h after 30 min preclearing with protein A/protein G Dynabeads (Invitrogen). Chromatin without any antibodies or with IgG antibodies was used as a negative control. The next day, Dynabeads were added to these samples and incubated for 2 h at 4 °C, which were later collected and washed with several buffers and finally eluted. Pulled-down DNA was purified using phenol-chloroform-isoamyl alcohol (25:24:1, Sigma) after reverse cross-linking of the eluted chromatin. Purified DNA was used as a template for qPCR to quantify the changes in binding of JMJD2D to its targets, where primers specific to the gene promoters were used. Input served as a positive control, while IgG or no antibody served as a negative control.

### 2.11. Behavioural Tests

For behavioural parameters, video tracking of mice movements was carried out using Ethovision 3.1 (Noldus, The Netherlands), and tests to evaluate anxiety and depression-like phenotype were executed.

Tests for anxiety—the open field test (OFT), light and dark box test (L&DBT), and zero maze test (ZMT) are classic paradigms used for assessing anxiety-like behaviour in mice that use the ethological conflict between the tendencies of mice to explore the novel environment and to avoid brightly lit and unprotected open areas.

In OFT, mice were individually introduced into a brightly lit open arena that was virtually divided into a “central zone” and “peripheral zone” and their movements were tracked for 5 min. Anxiety was evaluated based on the percentage of time spent in the central zone to the total time spent in the open field box.

In the L&DBT, using a partition, one box was divided into an open, brightly lit compartment and a closed, dark subdivision, where both were connected through a small opening in the partition. Mice were individually introduced into the illuminated “light zone” of this box and their movements were tracked for 5 min. The percentage of time spent in the light zone compared to the total time spent in the arena was taken as a measure for assessing anxiety-like behaviour.

In the ZMT, mice were individually placed on the illuminated “open area” of the zero maze apparatus that comprises a circular maze divided into two open and two closed zones and was kept elevated at 100 cm above the ground. The movements of the mice were tracked for 5 min and percentage of time spent in both the open zones was calculated and used to evaluate anxiety.

Tail suspension test for depression—mice were individually suspended by their tails at 50 cm above the ground for 5 min. Depression was assessed as the measure of the total time of immobilization (absence of escape-oriented behaviours).

### 2.12. Generation of the Jmjd2d Overexpressing AAV2 Particles and Their Purification

*Jmjd2d* coding sequence was amplified from mouse cDNA library and cloned into AAV2-IRES-hrGFP vector. Production and purification of the viral particles were carried out using a helper-free triple transfection method in human embryonic kidney 293 (HEK-293) cells (American Type Culture Collection, Manassas, VA, USA) as described earlier [57,58,59].

### 2.13. Statistical Analysis

The graphical results are presented as means ± S.E.M. Data was statistically analysed using Student’s *t*-test (in two groups) and one-way ANOVA (in case of multiple groups) where differences with *p*-value ≤ 0.05 were considered as significant. We have used * *p* < 0.05, ** *p* < 0.01, *** *p* < 0.001, **** *p* < 0.0001. Note: See Supplementary Figure S1 for a summary of work flow.

## 3. Results

### 3.1. CSDS for 10 Days Leads to Depression-Like Phenotype in Adult C57BL/6 Mice

**Figure 1:**
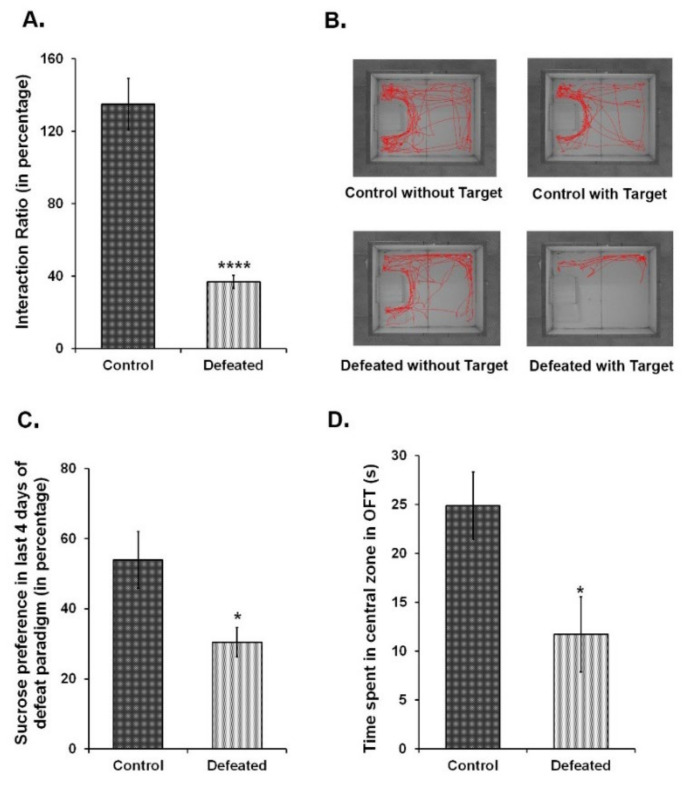
Ten days of chronic social defeat stress induce depression and anxiety-like phenotype in mice. (**A**) Interaction ratio {time spent in the interaction zone in presence of target CD1 (aggressor) mouse in (s)/time spent in the interaction zone in absence of target mouse in (s)} of the control and the defeated C57BL/6NCrl mice in the social interaction test. (**B**) The track plots of the control and defeated mouse in the presence and absence of the target CD1 mouse during 5 min of social interaction test. (**C**) Percentage consumption of 2% sucrose in the last four days of the chronic social defeat stress (CSDS) paradigm. (**D**) Time spent in (s) in the central zone during open field test (OFT) (*n* = 8–10/group). * *p* < 0.05 and **** *p* < 0.0001

To correlate the association between depression and related mood disorders with adult hippocampal neurogenesis, we independently subjected wild-type C57BL/6NCrl and Nestin-GFP transgenic mice to a 10-day CSDS paradigm [51,54]. Over this period of social subordination, experimental mice showed the characteristic submissive supine posture upon interacting with the aggressor. During the behavioural tests, these mice displayed social avoidance and anhedonia—two major hallmarks of depression in humans [27]. The defeated mice exhibited a significant reduction in social interaction with a non-familiar aggressor CD1 mouse (Figure 1A,B). The preference for a 2% sucrose solution, as compared to the plain water, was also significantly reduced in the defeated mice (Figure 1C). Furthermore, these mice had a pronounced anxiety-like phenotype as compared to the controls since they spent remarkably less time in the centre of the arena in the open field test (Figure 1D). Altogether, defeated mice showed the symptoms of both anxiety-like and depression-like phenotype.

### 3.2. CSDS-Induced Mood Disorders Are Associated with Attenuation of Neural Proliferation and Neurogenesis in Thel DG of the Hippocampus

**Figure 2:**
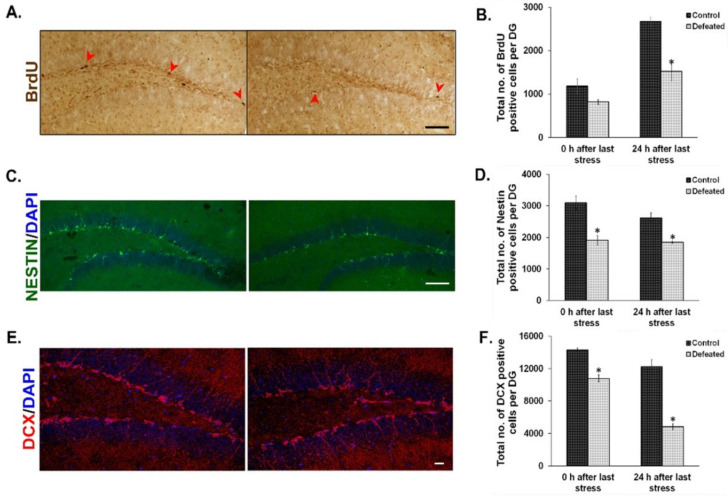
Social defeat stress leads to reduced neurogenesis in the hippocampal dentate gyrus (DG). (**A**) Representative images from BrdU staining in the DG of control (left) and defeated (right) mouse after 24 h of last defeat. Scale bar represents 100 µm. (**B**) Quantitative analysis revealed a significant decrease in the number of BrdU positive cells in the DG of defeated mice as compared to the control mice at 24 h after the last stress episode. (*n* = 4–5 mice/group). (**C**) Representative images of Nestin-GFP positive cells in the DG of control (left) and defeated (right) mouse 24 h after the last stress. Scale bar represents 100 µm (**D**) Quantitative analysis revealed a significant decrease in the Nestin-GFP positive cells in the DG of the defeated mice sacrificed immediately or after 24 h of the last stress (*n* = 5–6/group). (**E**) Representative images of Doublecortin (DCX) positive cells in the DG of control (left) and defeated (right) mouse after 24 h of the last defeat episode. Scale bar represents 200 µm (**F**) The number of DCX positive cells is significantly reduced in the DG of the defeated mice sacrificed immediately and after 24 h of the last stress episode than the control mice (*n* = 4/group). * *p* < 0.05.

The effect of CSDS on cell proliferation in the DG has already been shown earlier by BrdU staining [36]. On similar lines, we injected BrdU intraperitoneally in the defeated and control mice at two time points (explained in the methodology section) and counted BrdU positive cells in their DG. We found a noticeable reduction in the number of BrdU positive cells in the defeated mice sacrificed at both the time points tested. However, the attenuation was more profound and significant in the mice that were sacrificed 24 h after the last stress (Figure 2A,B). To reconfirm the effect of chronic stress on the proliferative NPC/NSC population, we used Nestin-GFP transgenic mice as a tool and counted Nestin-positive cells in both the conditions [60]. Defeated mice sacrificed either immediately or 24 h after the last stress, showed a significant reduction in the number of Nestin-GFP positive cells in their DG as compared to the controls (Figure 2C,D). We also found a significant decrease in the number of DCX (Doublecortin) positive cells in the DG of the defeated mice, at both the time points, as compared to the controls (Figure 2E,F). The decrease in proliferating cells demonstrated by both exogenous (BrdU incorporation) and endogenous (quantification of Nestin positive cells) methods and reduction in neuronal precursors or immature neurons (DCX staining) suggest an overall decrease in neurogenesis upon CSDS.

### 3.3. Jumonji C Domain-Containing H3 Lysine-Specific Demethylases (JMJDs/KDM4s) Get Dysregulated in the DG of Defeated Mice and May Affect Neurogenesis

**Figure 3:**
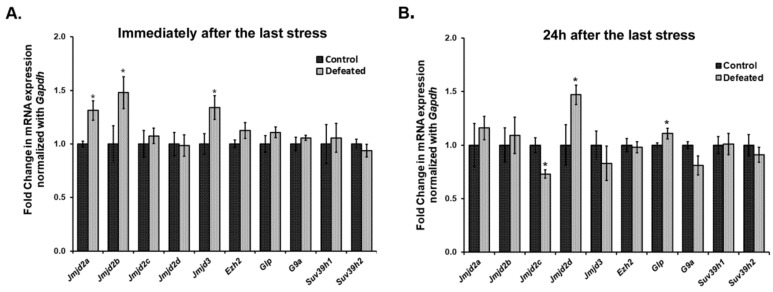
Ten days of CSDS causes dysregulation in the mRNA expression of a few of the histone lysine methylases and demethylases. mRNA expression levels of methylases and *Jmjd2/Jmjd3* family demethylases were analysed in the DG of the mice at both the time point using qPCR. (**A**) Two members of the *Jmjd2* family, *Jmjd2a*, and *Jmjd2b* along with *Jmjd3* exhibit a significantly higher mRNA expression in defeated mice as compared to controls, when mice were sacrificed immediately, i.e., 0 h after last defeat stress (*n* = 6–8/group). (**B**) There is a significant downregulation in the expression of *Jmjd2c* and upregulation in the expression of *Jmjd2d* in the defeated mice than the controls when mice were sacrificed 24 h after the last defeat event (*n* = 6–8/group). Further, one of the histone lysine methylases, *Glp*, also shows a significant increase in its mRNA levels in the defeated mice. * *p* < 0.05.

We quantified the mRNA expression levels of a few of the Jumonji C domain-containing lysine demethylases (*Jmjds*) and few lysine-specific methylases, in the DG of the defeated mice, sacrificed at 0 h (10th day) and 24 h (11th day) after the last stress event. Interestingly, we observed a highly dynamic expression pattern of *Jmjds* in both the conditions. Defeated mice, which were sacrificed 0 h after the last defeat episode, showed a significantly higher mRNA expression of *Jmjd2a*, *Jmjd2b*, and *Jmjd3* in their DG as compared to the controls (*p* < 0.05). The mRNA expression level for other *Jmjd2* family members and the methyltransferases was not altered (Figure 3A). On the other hand, when mRNA expression of these genes was analysed in DG samples of the mice sacrificed on the 11th day, we observed a different profile of these demethylases, where defeated mice displayed significantly higher levels of *Jmjd2d* and reduced levels of *Jmjd2c* as compared to the controls (*p* < 0.05). Additionally, one of the H3K9 methyltransferases, *Glp* also exhibited significantly increased mRNA expression in the defeated mice (*p* < 0.05). The other methylation regulators did not show any change in their expression (Figure 3B).

### 3.4. Inhibiting JMJD2 Family Demethylases Alters Proliferation of NPCs/NSCs in the Ex Vivo Neurosphere Culture

**Figure 4:**
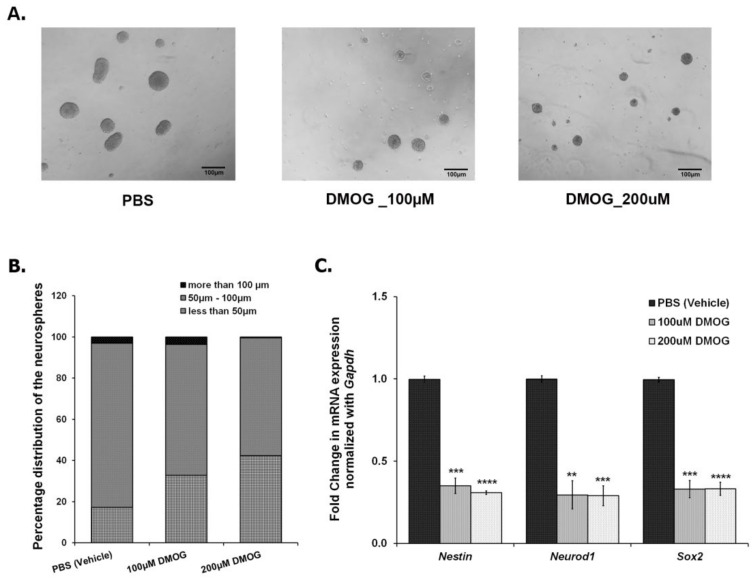
Treatment of NSCs/NPCs with DMOG, a pharmacologic inhibitor for JMJD2 family demethylases, resulted in a decrease in the proliferation of neurospheres (**A**) Representative images of the neurospheres treated with vehicle (PBS), 100 μM DMOG, and 200 μM DMOG. (**B**) Count of different sized neurospheres, taken after 6 days treatment with DMOG at different concentrations. (**C**) qPCR analysis shows a significant downregulation in the mRNA expression of *Nestin*, *Neurod1*, and *Sox2* in the DMOG treated samples as compared to the vehicle (PBS) treated ones. ** *p* < 0.01, *** *p* < 0.001, **** *p* < 0.0001

As shown in Figure 2, CSDS leads to a decrease in the number of proliferating cells in DG, thereby, reducing neurogenesis. In parallel, CSDS also affects the mRNA expression of *Jmjds* in DG of the defeated mice. To investigate an association of histone demethylases, if any, with the compromised neurogenesis, we used DMOG, which is an inhibitor of JMJD2 family proteins. We cultured the NSCs/NPCs derived from the hippocampus of the postnatal day 2 (PND2) mouse, treated with different doses of DMOG (100 μM, 200 μM, 400 μM, and 600 μM) daily for 6 days and performed the neurosphere assay. As the higher doses (400 μM and 600 μM) were toxic to the cells, we chose lower doses (100 μM and 200 μM) for our experiments. After 24 h of the last dose (7th day), we measured the diameter of the neurospheres and found a significant reduction in the number of large neurospheres (>100 μm) with DMOG treatment as compared to the vehicle (PBS) treatment (Figure 4A,B). However, there was no significant difference in the total number of neurospheres between both the groups. We also quantified the expression of a few of the proliferating markers (*Nestin*, *Neurod1*, and *Sox2*) and found a significant downregulation of their mRNA expression upon DMOG treatment (Figure 4C). Our results suggest that inhibition of JMJD2 family histone demethylases affects the proliferation of NPCs/NSCs, thereby reducing the size of the neurospheres.

### 3.5. Jmjds Get Dysregulated When Proliferating Neurospheres Are Subjected to Differentiation

**Figure 5:**
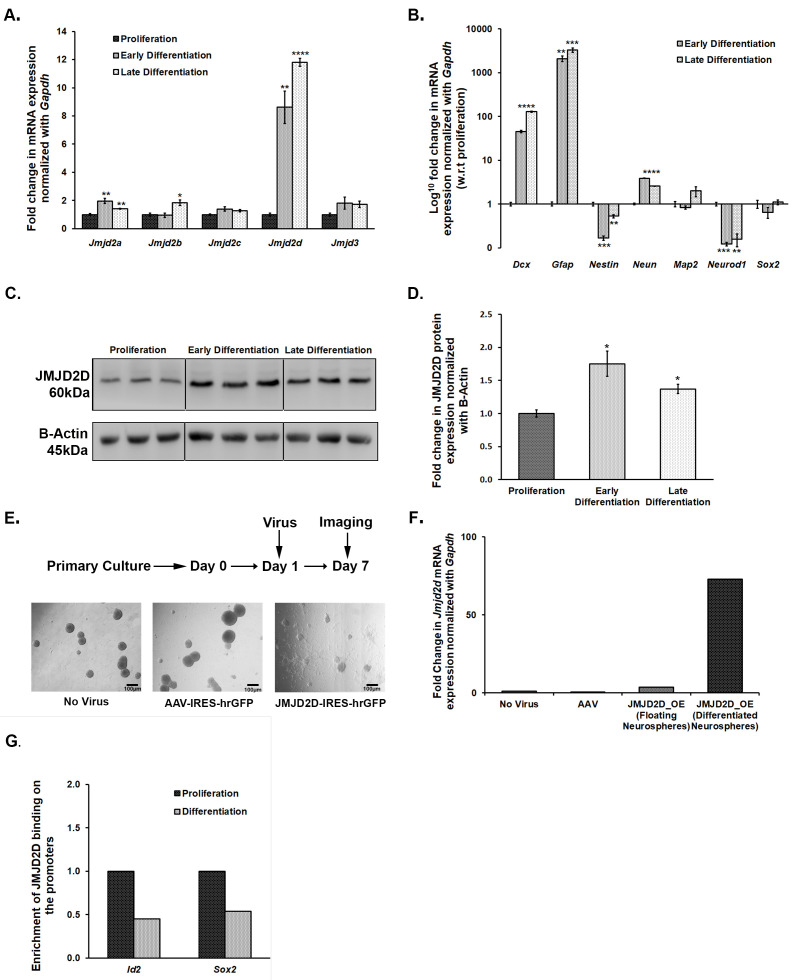
JMJD2D shows a critical role in regulating the proliferation and differentiation of NSCs/NPCs in the *ex vivo* primary neurosphere culture. (**A**) *Jmjd2a*, *Jmjd2b*, and *Jmjd2d* show a significant increase in their mRNA expression when proliferating neurospheres are subjected to differentiation. (**B**) mRNA expression pattern of the specific makers validates our proliferation-differentiation model. (**C**,**D**) JMJD2D also showed a significant upregulation at protein level upon differentiation. (**E**) Representative images of the neurosphere culture taken after 7 days of viral infection suggest differentiation of the NSCs/NPCs when they were infected with JMJD2D expressing AAV particles. (**F**) Significantly higher *Jmjd2d* mRNA expression in the differentiated population confirms that overexpression of *Jmjd2d* induces differentiation. (**G**) ChIP-qPCR analysis suggests that binding of JMJD2D decreases on *Sox2* and *Id2* promoters upon differentiation, which promotes differentiation. * *p* < 0.05, ** *p* < 0.01, *** *p* < 0.001, **** *p* < 0.0001.

As treatment with DMOG affected the size of neurospheres, we speculated the role of JMJDs in the growth and maintenance of NSCs/NPCs in culture conditions. For this, we subjected proliferating NSCs/NPCs to differentiation and mapped mRNA expression levels of *Jmjd2* family members and *Jmjd3* at early (day 2) and late (day 5) time-points using qPCR. Differentiation led to an increase in the expression of all these members except *Jmjd2c* and *Jmjd3* (Figure 5A). Interestingly, *Jmjd2d* showed almost an 8–10 fold increase in its mRNA expression upon differentiation (Figure 5A). Its increase in expression was validated at the protein level as well, albeit the increase was not so pronounced (Figure 5C,D). The expression pattern of a few of the known markers for NSC/NPC proliferation and differentiation was also tested to confirm that the culture conditions were standard (Figure 5B).

### 3.6. Overexpression of Jmjd2d Induces Differentiation of Neurospheres

We reasoned that the adverse effect of DMOG on the proliferation of neurospheres is probably through inhibiting the JMJD2 family of histone lysine demethylases. We have also shown the dysregulation of these *Jmjds* when proliferating neurospheres are subjected to differentiation. Thus, here, we attempted to specifically evaluate the role of one of the members, JMJD2D, which is upregulated upon differentiation of NPCs/NSCs and also in the DG of defeated mice. We incubated NSCs/NPCs with *Jmjd2d*-AAV2-IRES-hrGFP viral particles on the first day after passage to overexpress the *Jmjd2d* gene and allowed them to grow for 7 days. Cells lacking viral infection or those infected with control AAV2 particles formed round and large floating neurospheres, whereas cells infected with *Jmjd2d* overexpressing AAV2 viral particles could not form healthy neurospheres. Instead, most of the neurospheres settled down and formed projections, a typical hallmark of differentiation (Figure 5E). We could also spot a few floating neurospheres in this group that were probably formed by the cells which were not infected by *Jmjd2d* viral particles. For further validation, we collected both differentiated/flattened and floating population and quantified *Jmjd2d* mRNA levels by qPCR. Here we could find a much higher *Jmjd2d* expression in the flattened and differentiating population as compared to the round and floating ones (Figure 5F). Thus, *Jmjd2d* seems to play a significant role in inducing differentiation of the NPCs/NSCs.

### 3.7. JMJD2d Regulates Neural Stem or Progenitor Cell Differentiation by Regulating Transcription Factors Id2 and Sox2 Involved in Controlling Proliferation and Differentiation

Chromatin Immunoprecipitation (ChIP) followed by qPCR suggests a differential enrichment of JMJD2D on the promoters of some of the genes that play a critical role in the proliferation and differentiation of NSCs/NPCs. There was an enrichment of JMJD2D on the promoters of *Id2* (inhibitor of differentiation) and *Sox2* (SRY-Box Transcription Factor 2) genes in the proliferating NSCs/NPCs (Figure 5G). Following differentiation, a significant reduction in this enrichment was observed (Figure 5G). The attenuation in JMJD2D binding on these promoter regions would likely prevent the removal of the repressive methylation mark, H3K9me2, which may inhibit the expression of *Sox2* and *Id2* in the differentiating cells. Preventing the transcriptional activation of the gene that inhibits differentiation (*Id2*) could thus induce differentiation. *Sox2* level, if high, maintains the proliferation stage and stemness. The decrease in the enrichment of JMJD2D on its promoter can result in increased methylation and repression, leading to differentiation.

### 3.8. Administration of DMOG Induces Anxiety-Like and Depression-Like Phenotype in Mice without Any Exposure to Stress

**Figure 6:**
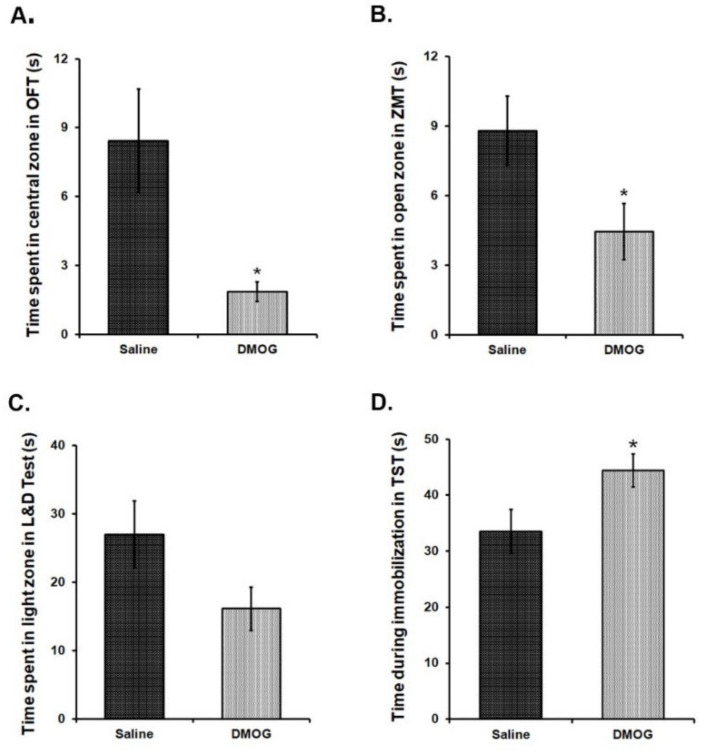
10 days chronic DMOG administration induced anxiety and depression-like phenotype in mice without any stress exposure. DMOG administered mice display an anxiety-like phenotype as they spend significantly lesser time in the central zone of the open field box (**A**), open zone of the zero maze (**B**), and light zone of the light and dark box (**C**) as compared to the vehicle (Saline) injected mice. (**D**) DMOG-injected mice spend significantly more time being immobilized in the tail suspension test, suggesting a depression-like phenotype. (*n* = 9–11/group). * *p* < 0.05.

We intraperitoneally (i. p.) administered DMOG in 6-8 weeks old C57BL/6NCrl mice at a dose of 40 mg/kg body weight, daily for 10 consecutive days. Control mice received the injections of an equal volume of saline for the same period. To assess anxiety-like and depression-like phenotype in these mice at the end of 10 days, we conducted a battery of behavioural tests for the next four days in these mice. Mice, administered with DMOG, spent significantly less time in exploring the brightly lit, central area in the open field test as compared to the saline administered controls (Figure 6A). Likewise, these mice also spent relatively less time in the open zones than the closed zones of the zero maze (Figure 6B) and in the brightly lit zone as compared to the covered dark zone of the light and dark box (Figure 6C). These results suggest higher anxiety-like phenotype in the mice treated with DMOG. When tested in the tail suspension test, DMOG-administered mice remained immobile significantly for a longer time than the saline-injected mice indicating the development of depression-like phenotype (*p* < 0.05) (Figure 6D). Therefore, we conclude that inhibiting the activity of all the JMJD2 family proteins induces anxiety and depression-like symptoms in mice even in the absence of physical or psychological stress.

## 4. Discussion

Depression and related psychiatric illnesses have complex etiopathology; however, recent molecular investigations have given a better insight into the underlying mechanisms [61,62]. Chronic stress precipitates psychiatric illnesses through profound effects on various brain regions, and these effects are often mediated by epigenetic regulatory mechanisms [63]. Response to chronic stress has been shown to be associated with a decrease in the proliferation of NSCs/NPCs in the subgranular zone of the hippocampal DG in adult rodent brain [64,65,66]. We have investigated the effect of chronic stress, using the CSDS paradigm, on adult hippocampal neurogenesis at two-time points—0 h, i.e., immediately after the last stress event and at 24 h after the last defeat stress. The results were in accordance with previously published results, i.e., decrease in BrdU-labelled proliferating cells and in Nestin-GFP positive cells in the DG immediately after the last stress event [67]. The number of both BrdU-labeled and Nestin-GFP positive cells were still less in the DG of the defeated mice 24 h after the last stress as compared with the control mice. This result is contrary to an earlier study [36] where a transient decrease in the proliferating cell population was reported to be normalized 24 h after the last stress. Additionally, earlier reports have shown a reduction in the newly differentiated neuronal population, as marked by Dcx (doublecortin) expression [37,68]. We also obtained similar results, where there was a significant decrease in the number of Dcx positive cells in the subgranular zone of DG of the defeated mice sacrificed at both of the time points.

Epigenetic mechanisms such as histone lysine acetylation and methylation have been implicated in mediating stress effects on the hippocampus and DG neurogenesis, in rodent models of mood and cognitive disorders [38,69]. It has been shown that elements of the epigenetic machinery, the histone acetyltransferases (HATs), histone deacetylases (HDACs), methyltransferases (KMTs), and demethylases (KDMs) work in coordination and are involved in the precise execution of various events of stem cell proliferation, their transition to various cell fates [70,71], and regulation of adult neurogenesis [38]. There are several reports on the dynamic interplay between numerous activating and repressing histone marks that regulate the proliferation and differentiation of NPCs/NSCs at their different stages. In one study, H3K27me3 specific demethylase, JMJD3 is reported to be essential for commitment to the neural fate [46]. Likewise, the demethylases regulating transcriptionally activating H3K4 methylation LSD1 and JARID1B, are also reported as regulators of NSC proliferation [45,72], and demethylases of PHF or KDM7 family are involved in neuronal differentiation [73,74].

KDM4/JMJD2 family members have been implicated in neuronal development too [75,76]. There are a few studies that specify their functions in cell proliferation and differentiation in non-neuronal cell types [77,78,79]. However, there has been no report on the role of the KDM4/JMJD2 family in regulating various events of neurogenesis in the adult brain. So far, the only study is by Cascante A. and colleagues, where KDM4A/C or JMJD2A/C has been shown to regulate rat cortical embryonic NSC differentiation by regulating glial fibrillary acidic protein (GFAP) [80]. Using a psychosocial defeat mouse model of depression and related mood disorders, we show a dynamic transcriptional dysregulation of KDM4/JMJD2 class demethylases in DG of the defeated mice sacrificed at two different time points. *Jmjd2a* and *Jmjd2b* showed an upregulation in the DG of the mice that were sacrificed immediately after the last defeat episode, i.e., at 0 h. *Jmjd3*, an H3K27 demethylase, which is actively involved in neural stem cell biology, also exhibited an upregulation in the DG of these mice. On the other hand, *Jmjd2c* was found downregulated whereas *Jmjd2d* was upregulated in the DG of the defeated mice that were sacrificed 24 h after the last defeat episode, as compared to the control mice. In addition to the proliferating NSCs/NPCs, DG comprises a heterogeneous population of immature and mature neurons and astrocytes, which made it technically challenging to dissect the role of JMJD2 class demethylases in proliferation and differentiation *in vivo*. Therefore, we used a simpler *ex vivo* neurosphere culture system derived from the NSCs/NPCs of the hippocampal DG from mouse neonates for this purpose.

Our *ex vivo* and *in vivo* findings support a role for the KDM4/JMJD2 family in mediating the effects of psychosocial stress on adult hippocampal neurogenesis. First, the quantitative analysis of mRNA expression levels suggested a significant dysregulation of *Jmjd2c* and *Jmjd2d* in the DG of defeated mice, 24 h after the last defeat stress, which indicated their potential role in the neurogenic niche in response to chronic stress. Second, the treatment of *ex vivo* neurosphere culture with DMOG, a pharmacologic inhibitor of the JMJD2 family of demethylases, exhibited a significant reduction in proliferation of the neurospheres. DMOG treated neurospheres also showed a decrease in the mRNA levels of the NSC/NPC proliferation markers, *Nestin* and *Sox2*, and early differentiation marker, *Neurod1*, indicating a direct effect on the turnover of NSCs. Our study was in accordance with a previous study [81] that also reported an anti-proliferative property of DMOG. This suggests that the inhibition of JMJD2/KDM4 family members is detrimental for the proliferation of neurospheres. However, to understand specifically which of the four members is crucial for the process, further investigation is needed.

The data on mRNA analysis using *ex vivo* neurosphere culture gave us better insight into the role of JMJD2 family, in particular *Jmjd2d*, in neural proliferation and differentiation. *Jmjd2d* showed a significant upregulation when proliferating NSCs/NPCs were subjected to differentiation. It was upregulated in the DG of the defeated mice as well, dissected 24 h after the last defeat episode. The inverse correlation between *Jmjd2d* expression and proliferation of NSCs/NPCs, both in the neurosphere culture and in the DG of the defeated mice, suggests its critical role in this process. This was validated when the overexpression of *Jmjd2d* in neurosphere culture attenuated their proliferation and induced differentiation. Our ChIP and qPCR experiments showed that JMJD2D is enriched on the promoters of *Id2* and *Sox2* during proliferation. Despite an increase in the level of JMJD2D during differentiation, its enrichment on the promoters of *Id2* and *Sox2* was found to decrease. We hypothesize that its enrichment would lead to demethylation of the transcriptionally repressive H3K9 methylation on promoter regions and hence keep these genes (*Id2* and *Sox2*) active during proliferation. JMJD2D upregulation during differentiation would lead to an increase in demethylation at H3K9, which could activate certain genes required for differentiation. In addition to acting on H3K9, it also targets H3K36, which is an active methylation mark. Its upregulation would result in the removal of H3K36 at several gene promoters which could eventually lead to the repression of specific genes necessary for proliferation. Finally, induction of anxiety and depression-like phenotype in mice, without any physical or psychological stress, upon chronic intraperitoneal DMOG administration, clearly indicates a significant role of the KDM4/JMJD2 family of histone lysine demethylases in mediating these effects. We assume that *in vivo* blockade of JMJD2 class members would inhibit demethylation at the transcriptionally repressive H3K9 methylation. This would lead to a decreased expression of several genes which may result in altered neurogliogenesis, hippocampal neuroglial plasticity, and ultimately, mood and behavior (which is reflected in mood disorders). A previous study from our lab also showed the behavioural effects of DMOG treatment on the mood in mice [49]; however, in the current study, the treatment was for a longer period and the behavioural outcomes were more pronounced.

DMOG is also used as a hypoxia mimetic agent, which could have an effect on the mood and behaviour of the animals [82,83]. However, we had shown in a previous study that despite uncoupling the effect of DMOG on hypoxia, it was able to increase H3K9me2 levels in the brain that can be explained by its inhibitory action on JMJD2/KDM4 family [55]. The behavioural outcome of DMOG administration in the present study could also be correlated with the DMOG-induced compromised proliferation and premature differentiation of NPCs/NSCs. Similarly, dysregulation and altered expression profiles of the JMJD2/KDM4 family of lysine demethylases in the mouse DG in response to CSDS would negatively affect hippocampal neurogenesis, eventually contributing to the development of anxiety and depression-like phenotype.

Thus, epigenetic regulators of the JMJD2/KDM4 family of lysine demethylases, in particular JMJD2D, appear to be critical mediators of the chronic stress effects on the neurogenesis in the CSDS-induced depression model. JMJD2D has been shown to interact with and regulate signalling pathways like Hedgehog signalling, hypoxia signalling, and Wnt signalling under different conditions [79,84,85]. Since all these signalling pathways have potential implications for neurogenesis [86,87,88], it would be interesting to investigate if this is one of the ways JMJD2D influences neurogenesis. Unravelling the role of JMJD2D and other family members in detail in the adult mouse brain might help in strategizing the therapeutic approach to treat affective disorders like anxiety and depression.

## Supplementary Materials

The following are available online at https://www.mdpi.com/2076-3425/10/11/833/s1, Figure S1: Summary of the work flow.

## References

[1] De Kloet E.R., Joëls M., Holsboer F. (2005). Stress and the brain: From adaptation to disease. Nat. Rev. Neurosci..

[2] Kendler K.S., Karkowski L.M., Prescott C.A. (1999). Causal Relationship between Stressful Life Events and the Onset of Major Depression. Am. J. Psychiatry.

[3] Vos T., Abajobir A.A., Abbafati C., Abbas K.M., Abate K.H., Abd-Allah F., Abdulle A.M., Abebo T.A., Abera S.F., Aboyans V. (2017). Global, regional, and national incidence, prevalence, and years lived with disability for 328 diseases and injuries for 195 countries, 1990–2016: A systematic analysis for the Global Burden of Disease Study 2016. Lancet.

[4] Al-Harbi K.S. (2012). Treatment-resistant depression: Therapeutic trends, challenges, and future directions. Patient Prefer. Adherence.

[5] Khawam E.A., Laurencic G., Malone D.A. (2006). Side effects of antidepressants: An overview. Clevel. Clin. J. Med..

[6] Masand P.S., Gupta S. (2002). Long-Term Side Effects of Newer-Generation Antidepressants: SSRIS, Venlafaxine, Nefazodone, Bupropion, and Mirtazapine. Ann. Clin. Psychiatry.

[7] Altman J. (1962). Autoradiographic study of degenerative and regenerative proliferation of neuroglia cells with tritiated thymidine. Exp. Neurol..

[8] Altman J. (1963). Autoradiographic investigation of cell proliferation in the brains of rats and cats. Anat. Rec..

[9] Cameron H.A., Woolley C.S., McEwen B.S., Gould E. (1993). Differentiation of newly born neurons and glia in the dentate gyrus of the adult rat. Neuroscience.

[10] Lucassen P.J., Oomen C.A., Schouten M., Encinas J.M., Fitzsimons C.P. (2016). Chapter 8—Adult Neurogenesis, Chronic Stress and Depression. Adult Neurogenesis in the Hippocampus.

[11] Moreno-Jiménez E.P., Flor-García M., Terreros-Roncal J., Rábano A., Cafini F., Pallas-Bazarra N., Ávila J., Llorens-Martín M. (2019). Adult hippocampal neurogenesis is abundant in neurologically healthy subjects and drops sharply in patients with Alzheimer’s disease. Nat. Med..

[12] Seki T. (2020). Understanding the real state of human adult hippocampal neurogenesis from studies of rodents and non-human primates. Front. Neurosci..

[13] Gage F.H., van Praag H., Kempermann G. (1999). Running increases cell proliferation and neurogenesis in the adult mouse dentate gyrus. Nat. Neurosci..

[14] Lucassen P.J., Meerlo P., Naylor A.S., van Dam A.M., Dayer A.G., Fuchs E., Oomen C.A., Czéh B. (2010). Regulation of adult neurogenesis by stress, sleep disruption, exercise and inflammation: Implications for depression and antidepressant action. Eur. Neuropsychopharmacol..

[15] Madsen T.M., Treschow A., Bengzon J., Bolwig T.G., Lindvall O., Tingström A. (2000). Increased neurogenesis in a model of electroconvulsive therapy. Biol. Psychiatry.

[16] Mirescu C., Peters J.D., Noiman L., Gould E. (2006). Sleep deprivation inhibits adult neurogenesis in the hippocampus by elevating glucocorticoids. Proc. Natl. Acad. Sci. USA.

[17] Schoenfeld T.J., Gould E. (2013). Differential Effects of Stress and Glucocorticoids on Adult Neurogenesis.

[18] Chakravarty S., Pathak S.S., Maitra S., Khandelwal N., Chandra K.B., Kumar A. (2014). Epigenetic regulatory mechanisms in stress-induced behavior. International Review of Neurobiology.

[19] Krishnan V., Nestler E.J. (2010). Linking Molecules to Mood: New Insight Into the Biology of Depression. Am. J. Psychiatry.

[20] McEwen B.S. (1999). Stress and hippocampal plasticity. Ann. Rev. Neurosci..

[21] Tsankova N.M., Renthal W., Kumar A., Nestler E.J. (2007). Epigenetic regulation in psychiatric disorders. Nat. Rev. Neurosci..

[22] Berton O., McClung C.A., Dileone R.J., Krishnan V., Renthal W., Russo S.J., Graham D., Tsankova N.M., Bolanos C.A., Rios M. (2006). Essential role of BDNF in the mesolimbic dopamine pathway in social defeat stress. Science.

[23] Campbell S., MacQueen G. (2004). The role of the hippocampus in the pathophysiology of major depression. J. Psychiatry Neurosci..

[24] Campbell S., Marriott M., Nahmias C., MacQueen G.M. (2004). Lower hippocampal volume in patients suffering from depression: A meta-analysis. Am. J. Psychiatry.

[25] Khandelwal N., Dey S.K., Chakravarty S., Kumar A. (2019). miR-30 Family miRNAs Mediate the Effect of Chronic Social Defeat Stress on Hippocampal Neurogenesis in Mouse Depression Model. Front. Mol. Neurosci..

[26] Vialou V., Feng J., Robison A.J., Nestler E.J. (2013). Epigenetic mechanisms of depression and antidepressant action. Annu. Rev. Pharmacol. Toxicol..

[27] Tsankova N.M., Berton O., Renthal W., Kumar A., Neve R.L., Nestler E.J. (2006). Sustained hippocampal chromatin regulation in a mouse model of depression and antidepressant action. Nat. Neurosci..

[28] Tsankova N.M., Kumar A., Nestler E.J. (2004). Histone Modifications at Gene Promoter Regions in Rat Hippocampus after Acute and Chronic Electroconvulsive Seizures. J. Neurosci..

[29] Jun H., Mohammed Qasim Hussaini S., Rigby M.J., Jang M.-H. (2012). Functional role of adult hippocampal neurogenesis as a therapeutic strategy for mental disorders. Neural Plast..

[30] Rotheneichner P., Lange S., O’Sullivan A., Marschallinger J., Zaunmair P., Geretsegger C., Aigner L., Couillard-Despres S. (2014). Hippocampal neurogenesis and antidepressive therapy: Shocking relations. Neural Plast..

[31] Sun H., Kennedy P.J., Nestler E.J. (2013). Epigenetics of the depressed brain: Role of histone acetylation and methylation. Neuropsychopharmacology.

[32] Wilkinson M.B., Xiao G., Kumar A., LaPlant Q., Renthal W., Sikder D., Kodadek T.J., Nestler E.J. (2009). Imipramine treatment and resiliency exhibit similar chromatin regulation in the mouse nucleus accumbens in depression models. J. Neurosci..

[33] Kenworthy C.A., Sengupta A., Luz S.M., Ver Hoeve E.S., Meda K., Bhatnagar S., Abel T. (2014). Social defeat induces changes in histone acetylation and expression of histone modifying enzymes in the ventral hippocampus, prefrontal cortex, and dorsal raphe nucleus. Neuroscience.

[34] Mallei A., Ieraci A., Popoli M. (2019). Chronic social defeat stress differentially regulates the expression of BDNF transcripts and epigenetic modifying enzymes in susceptible and resilient mice. World J. Biol. Psychiatry.

[35] Hollis F., Kabbaj M. (2014). Social defeat as an animal model for depression. ILAR J..

[36] Lagace D.C., Donovan M.H., DeCarolis N.A., Farnbauch L.A., Malhotra S., Berton O., Nestler E.J., Krishnan V., Eisch A.J. (2010). Adult hippocampal neurogenesis is functionally important for stress-induced social avoidance. Proc. Natl. Acad. Sci. USA.

[37] Yohn C.N., Ashamalla S.A., Bokka L., Gergues M.M., Garino A., Samuels B.A. (2019). Social instability is an effective chronic stress paradigm for both male and female mice. Neuropharmacology.

[38] Hsieh J., Nakashima K., Kuwabara T., Mejia E., Gage F.H. (2004). Histone deacetylase inhibition-mediated neuronal differentiation of multipotent adult neural progenitor cells. Proc. Natl. Acad. Sci. USA.

[39] Jawerka M., Colak D., Dimou L., Spiller C., Lagger S., Montgomery R.L., Olson E.N., Wurst W., Göttlicher M., Götz M. (2010). The specific role of histone deacetylase 2 in adult neurogenesis. Neuron Glia Biol..

[40] Mateus-Pinheiro A., Pinto L., Sousa N. (2011). Epigenetic (de)regulation of adult hippocampal neurogenesis: Implications for depression. Clin. Epigenet..

[41] Milne T.A., McEwen B.S., Hunter R.G., Pfaff D.W., McCarthy K.J. (2009). Regulation of hippocampal H3 histone methylation by acute and chronic stress. Proc. Natl. Acad. Sci. USA.

[42] Amente S., Lania L., Majello B. (2013). The histone LSD1 demethylase in stemness and cancer transcription programs. Biochim. Biophys. Acta BBA Gene Regul. Mech..

[43] Wu H., Sun Y.E. (2006). Epigenetic Regulation of Stem Cell Differentiation. Pediatr. Res..

[44] Murao N., Noguchi H., Nakashima K. (2016). Epigenetic regulation of neural stem cell property from embryo to adult. Neuroepigenetics.

[45] Sun G., Alzayady K., Stewart R., Ye P., Yang S., Li W., Shi Y. (2010). Histone demethylase LSD1 regulates neural stem cell proliferation. Mol. Cell. Biol..

[46] Burgold T., Spreafico F., De Santa F., Totaro M.G., Prosperini E., Natoli G., Testa G. (2008). The Histone H3 Lysine 27-Specific Demethylase Jmjd3 Is Required for Neural Commitment. PLoS ONE.

[47] Suri D., Veenit V., Sarkar A., Thiagarajan D., Kumar A., Nestler E.J., Galande S., Vaidya V.A. (2013). Early stress evokes age-dependent biphasic changes in hippocampal neurogenesis, BDNF expression, and cognition. Biol. Psychiatry.

[48] Krishnan V., Han M.H., Graham D.L., Berton O., Renthal W., Russo S.J., Laplant Q., Graham A., Lutter M., Lagace D.C. (2007). Molecular adaptations underlying susceptibility and resistance to social defeat in brain reward regions. Cell.

[49] Pathak S.S., Maitra S., Chakravarty S., Kumar A. (2017). Histone Lysine Demethylases of JMJD2 or KDM4 Family are Important Epigenetic Regulators in Reward Circuitry in the Etiopathology of Depression. Neuropsychopharmacology.

[50] Veeraiah P., Noronha J.M., Maitra S., Bagga P., Khandelwal N., Chakravarty S., Kumar A., Patel A.B. (2014). Dysfunctional Glutamatergic and γ-Aminobutyric Acidergic Activities in Prefrontal Cortex of Mice in Social Defeat Model of Depression. Biol. Psychiatry.

[51] Chakravarty S., Maitra S., Reddy R.G., Das T., Jhelum P., Kootar S., Rajan W.D., Samanta A., Samineni R., Pabbaraja S. (2015). A novel natural product inspired scaffold with robust neurotrophic, neurogenic and neuroprotective action. Sci. Rep..

[52] Chakravarty S., Reddy B.R., Sudhakar S.R., Saxena S., Das T., Meghah V., Brahmendra Swamy C.V., Kumar A., Idris M.M. (2013). Chronic Unpredictable Stress (CUS)-Induced Anxiety and Related Mood Disorders in a Zebrafish Model: Altered Brain Proteome Profile Implicates Mitochondrial Dysfunction. PLoS ONE.

[53] Kulkarni V.A., Jha S., Vaidya V.A. (2002). Depletion of norepinephrine decreases the proliferation, but does not influence the survival and differentiation, of granule cell progenitors in the adult rat hippocampus. Eur. J. Neurosci..

[54] Yanpallewar S.U., Fernandes K., Marathe S.V., Vadodaria K.C., Jhaveri D., Rommelfanger K., Ladiwala U., Jha S., Muthig V., Hein L. (2010). Alpha2-adrenoceptor blockade accelerates the neurogenic, neurotrophic, and behavioral effects of chronic antidepressant treatment. J. Neurosci..

[55] Chakravarty S., Jhelum P., Bhat U.A., Rajan W.D., Maitra S., Pathak S.S., Patel A.B., Kumar A. (2017). Insights into the epigenetic mechanisms involving histone lysine methylation and demethylation in ischemia induced damage and repair has therapeutic implication. Biochim. Biophys. Acta BBA Mol. Bas. Dis..

[56] Kumar A., Choi K.-H., Renthal W., Tsankova N.M., Theobald D.E.H., Truong H.-T., Russo S.J., Laplant Q., Sasaki T.S., Whistler K.N. (2005). Chromatin remodeling is a key mechanism underlying cocaine-induced plasticity in striatum. Neuron.

[57] Hommel J.D., Sears R.M., Georgescu D., Simmons D.L., DiLeone R.J. (2003). Local gene knockdown in the brain using viral-mediated RNA interference. Nat. Med..

[58] Mukherjee S., Coque L., Cao J.-L., Kumar J., Chakravarty S., Asaithamby A., Graham A., Gordon E., Enwright J.F., DiLeone R.J. (2010). Knockdown of Clock in the Ventral Tegmental Area Through RNA Interference Results in a Mixed State of Mania and Depression-Like Behavior. Biol. Psychiatry.

[59] Zolotukhin S., Byrne B.J., Mason E., Zolotukhin I., Potter M., Chesnut K., Summerford C., Samulski R.J., Muzyczka N. (1999). Recombinant adeno-associated virus purification using novel methods improves infectious titer and yield. Gene Ther..

[60] Kapoor R., Desouza L.A., Nanavaty I.N., Kernie S.G., Vaidya V.A. (2012). Thyroid Hormone Accelerates the Differentiation of Adult Hippocampal Progenitors. J. Neuroendocrinol..

[61] Fox M.E., Lobo M.K. (2019). The molecular and cellular mechanisms of depression: A focus on reward circuitry. Mol. Psychiatry.

[62] Krishnan V., Nestler E.J. (2008). The molecular neurobiology of depression. Nature.

[63] Bagot R.C., Labonté B., Peña C.J., Nestler E.J. (2014). Epigenetic signaling in psychiatric disorders: Stress and depression. Dialogues Clin. Neurosci..

[64] Eisch A.J., Petrik D. (2012). Depression and hippocampal neurogenesis: A road to remission?. Science.

[65] Joels M., Karst H., Krugers H.J., Lucassen P.J. (2007). Chronic stress: Implications for neuronal morphology, function and neurogenesis. Front. Neuroendocrinol..

[66] Pittenger C., Duman R.S. (2008). Stress, Depression, and Neuroplasticity: A Convergence of Mechanisms. Neuropsychopharmacology.

[67] Schmidt H.D., Duman R.S. (2007). The role of neurotrophic factors in adult hippocampal neurogenesis, antidepressant treatments and animal models of depressive-like behavior. Behav. Pharmacol..

[68] Van Bokhoven P., Oomen C.A., Hoogendijk W.J.G., Smit A.B., Lucassen P.J., Spijker S. (2011). Reduction in hippocampal neurogenesis after social defeat is long-lasting and responsive to late antidepressant treatment. Eur. J. Neurosci..

[69] Jessberger S., Nakashima K., Clemenson G.D., Mejia E., Mathews E., Ure K., Ogawa S., Sinton C.M., Gage F.H., Hsieh J. (2007). Epigenetic modulation of seizure-induced neurogenesis and cognitive decline. J. Neurosci..

[70] Hirabayashi Y., Suzki N., Tsuboi M., Endo T.A., Toyoda T., Shinga J., Koseki H., Vidal M., Gotoh Y. (2009). Polycomb limits the neurogenic competence of neural precursor cells to promote astrogenic fate transition. Neuron.

[71] Pasini D., Bracken A.P., Hansen J.B., Capillo M., Helin K. (2007). The polycomb group protein Suz12 is required for embryonic stem cell differentiation. Mol. Cell. Biol..

[72] Schmitz S.U., Albert M., Malatesta M., Morey L., Johansen J.V., Bak M., Tommerup N., Abarrategui I., Helin K. (2011). Jarid1b targets genes regulating development and is involved in neural differentiation. EMBO J..

[73] Huang C., Xiang Y., Chen C.D., Zhu Q., Chen J., Jing N., Zhang T. (2010). The dual histone demethylase KDM7A promotes neural induction in early chick embryos. Dev. Dyn..

[74] Qiu J., Shi G., Jia Y., Li J., Wu M., Li J., Dong S., Wong J. (2010). The X-linked mental retardation gene PHF8 is a histone demethylase involved in neuronal differentiation. Cell Res..

[75] Fueyo R., García M.A., Martínez-balbás M.A. (2015). Jumonji family histone demethylases in neural development. Cell Tissue Res..

[76] Fujiwara K., Fujita Y., Kasai A., Onaka Y., Hashimoto H., Okada H., Yamashita T. (2016). Deletion of JMJD2B in neurons leads to defective spine maturation, hyperactive behavior and memory deficits in mouse. Transl. Psychiatry.

[77] Agger K., Nishimura K., Miyagi S., Messling J.E., Rasmussen K.D., Helin K. (2019). The KDM4/JMJD2 histone demethylases are required for hematopoietic stem cell maintenance. Blood.

[78] Kurozumi A., Nakano K., Yamagata K., Okada Y., Nakayamada S., Tanaka Y. (2019). IL-6 and sIL-6R induces STAT3-dependent differentiation of human VSMCs into osteoblast-like cells through JMJD2B-mediated histone demethylation of RUNX2. Bone.

[79] Peng K., Kou L., Yu L., Bai C., Li M., Mo P., Li W., Yu C. (2019). Histone Demethylase JMJD2D Interacts with β-Catenin to Induce Transcription and Activate Colorectal Cancer Cell Proliferation and Tumor Growth in Mice. Gastroenterology.

[80] Cascante A., Klum S., Biswas M., Antolin-Fontes B., Barnabé-Heider F., Hermanson O. (2014). Gene-specific methylation control of H3K9 and H3K36 on neurotrophic BDNF versus astroglial GFAP genes by KDM4A/C regulates neural stem cell differentiation. J. Mol. Biol..

[81] Milosevic J., Adler I., Manaenko A., Schwarz S.C., Walkinshaw G., Arend M., Flippin L.A., Storch A., Schwarz J. (2009). Non-hypoxic stabilization of hypoxia-inducible factor alpha (HIF-alpha): Relevance in neural progenitor/stem cells. Neurotox. Res..

[82] Davis C.K., Jain S.A., Bae O.N., Majid A., Rajanikant G.K. (2019). Hypoxia mimetic agents for ischemic stroke. Front. Cell Dev. Biol..

[83] Zhao F., Yang J., Cui R. (2017). Effect of Hypoxic Injury in Mood Disorder. Neural Plasticity.

[84] Peng K., Zhuo M., Li M., Chen Q., Mo P., Yu C. (2020). Histone demethylase JMJD2D activates HIF1 signaling pathway via multiple mechanisms to promote colorectal cancer glycolysis and progression. Oncogene.

[85] Zhuo M., Chen W., Shang S., Guo P., Peng K., Li M., Mo P., Zhang Y., Qiu X., Li W. (2020). Inflammation-induced JMJD2D promotes colitis recovery and colon tumorigenesis by activating Hedgehog signaling. Oncogene.

[86] Qi C., Zhang J., Chen X., Wan J., Wang J., Zhang P., Liu Y. (2017). Hypoxia stimulates neural stem cell proliferation by increasing HIF-1α expression and activating Wnt/β-catenin signaling. Cell. Mol. Biol..

[87] Varela-Nallar L., Inestrosa N.C. (2013). Wnt signaling in the regulation of adult hippocampal neurogenesis. Front. Cell. Neurosci..

[88] Yao P.J., Petralia R.S., Mattson M.P. (2016). Sonic Hedgehog Signaling and Hippocampal Neuroplasticity. Trends Neurosci..

