# Supplementary material for: Histone Lysine Demethylase JMJD2D/KDM4D and Family Members Mediate Effects of Chronic Social Defeat Stress on Mouse Hippocampal Neurogenesis and Mood Disorders
Source: Brain Sci. 2020 Nov 9;10(11):833. doi: 10.3390/brainsci10110833 (PMC7695311; doi:10.3390/brainsci10110833)
Supplement: Supplementary file 1 [file brainsci-10-00833-s001.pdf]

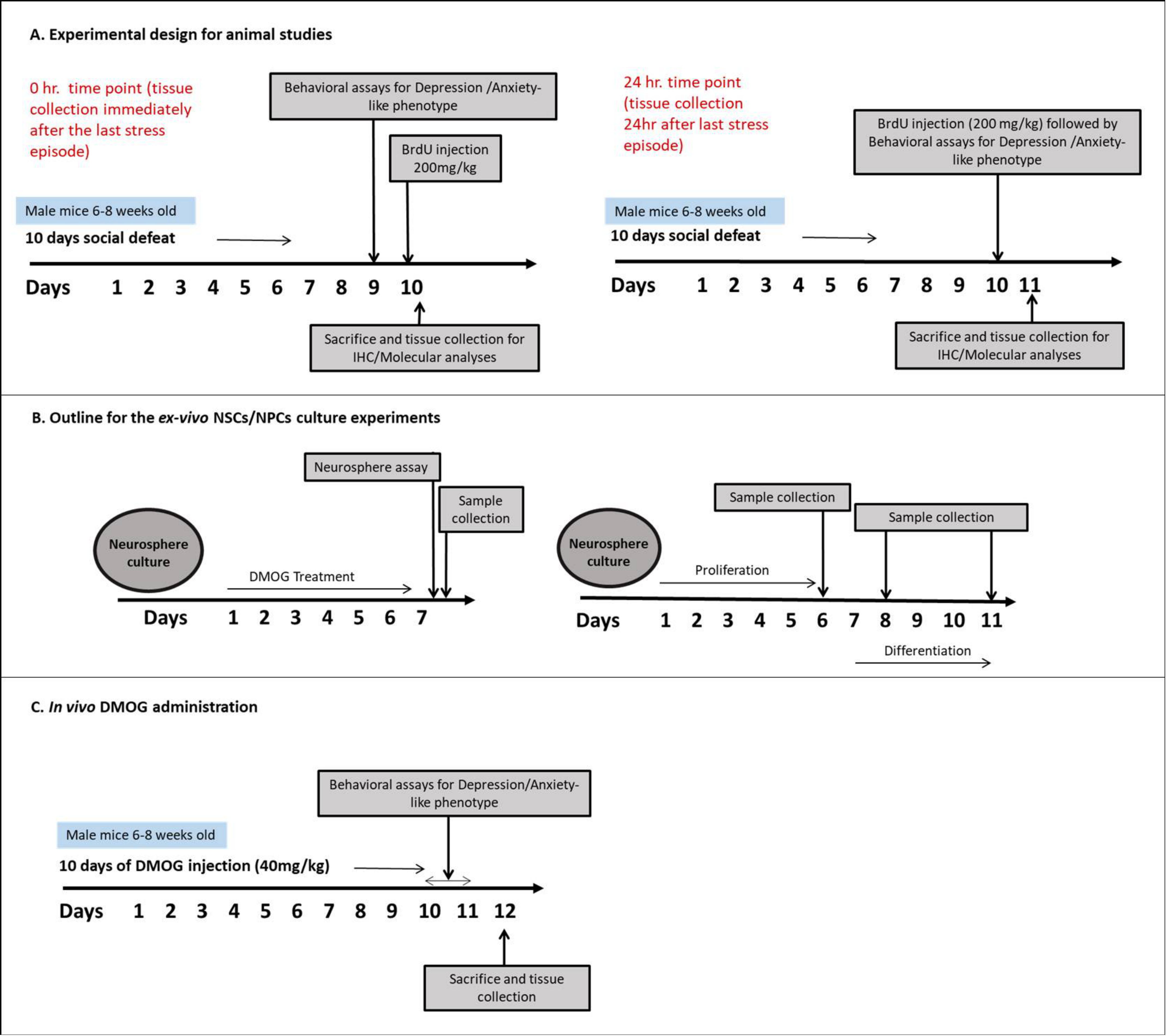
A. Experimental design for animal studies
0 hr. time point (tissue collection immediately after the last stress episode)
Behavioral assays for Depression /Anxiety-like phenotype
BrdU injection 200mg/kg
Male mice 6-8 weeks old
10 days social defeat
Days 1 2 3 4 5 6 7 8 9 10
Sacrifice and tissue collection for IHC/Molecular analyses
24 hr. time point (tissue collection 24hr after last stress episode)
BrdU injection (200 mg/kg) followed by Behavioral assays for Depression /Anxiety-like phenotype
Male mice 6-8 weeks old
10 days social defeat
Days 1 2 3 4 5 6 7 8 9 10 11
Sacrifice and tissue collection for IHC/Molecular analyses
B. Outline for the ex-vivo NSCs/NPCs culture experiments
Neurosphere assay
Sample collection
Neurosphere culture
DMOG Treatment
Days 1 2 3 4 5 6 7
Sample collection
Sample collection
Neurosphere culture
Proliferation
Days 1 2 3 4 5 6 7 8 9 10 11
Differentiation
C. In vivo DMOG administration
Behavioral assays for Depression/Anxiety-like phenotype
Male mice 6-8 weeks old
10 days of DMOG injection (40mg/kg)
Days 1 2 3 4 5 6 7 8 9 10 11 12
Sacrifice and tissue collection
